# Which is the best intrauterine insemination timing choice following exogenous hCG administration during ovulation induction by using clomiphene citrate treatment? A retrospective study

**DOI:** 10.1186/s40064-016-2992-9

**Published:** 2016-08-09

**Authors:** Omer Hamid Yumusak, Serkan Kahyaoglu, Meryem Kuru Pekcan, Esra Isci, Şebnem Ozyer, Mahmut Nedim Cicek, Yasemin Tasci, Salim Erkaya

**Affiliations:** Department of Reproductive Endocrinology, Zekai Tahir Burak Women’s Health Education and Research Hospital, Hamamonu-Altindag, Ankara Turkey

**Keywords:** Clinical pregnancy, Clomiphene citrate, Infertility, Intrauterine insemination

## Abstract

**Objective:**

To evaluate the impact of intrauterine insemination timing performed 24 or 36 h later following ovulation trigger on clinical pregnancy rate during ovulation induction with clomiphene citrate among infertile women was the objective of this study.

**Methods:**

The medical records of 280 infertile patients who have underwent ovulation induction by using clomiphene citrate have been evaluated and cycle outcomes of the patients have been investigated specifically based on the timing of intrauterine insemination during the treatment cycle.

**Results:**

The clinical pregnancy rate of the study group based on the timing of intrauterine insemination (24 vs. 36 h following hCG trigger) was found to be similar regardless of infertility type. The cycle day of which hCG trigger has been performed was found to be significantly longer for patients who have achieved clinical pregnancy than patients who have not got pregnant following the treatment cycle. Dominant follicle diameter has not been found to affect clinical pregnancy rate during treatment cycles with clomiphene citrate.

**Conclusions:**

In this study, intrauterine insemination timing did not affect the cycle outcomes whether the procedure has been performed 24 or 36 h later following ovulation trigger with exogenous hCG utilization. The longer period of treatment cycle during ovulation induction with clomiphene citrate resulted with higher clinical pregnancy rate. Intrauterine insemination can be done successfully at either 24 or 36 h after hCG in clomiphene citrate stimulated cycles. This will allow more flexibility and convenience for both physicians and patients, especially during weekends.

## Background

Controlled ovarian stimulation (COS) with intrauterine insemination (IUI) is a widely used fertility treatment for couples to improve pregnancy rates with mild male factor, unexplained infertility, cervical factor, anovulation, minimal and mild endometriosis (Goverde et al. 2000). It is simple, relatively less invazive and expensive procedure than other forms of assisted reproductive technologies (Dodson and Haney 1991).

Many different time intervals have been suggested in ovulation induction cycles after hCG injection for the management of infertility so there has been uncertainty in the correct timing of insemination. In 1970’s Edwards and Steptoe showed that ovulation began at 36–38 h after an injection of hCG if the follicular development was adequate (Edwards and Steptoe 1974). However Yang et al. showed that intrauterine insemination can be done at any time between 1 and 48 h after hCG injection without affecting significantly pregnancy outcomes. This flexibility in COH-IUI cycles provides more convenience for both patients and clinic staff, especially during weekends (Yang et al. 2008). According to World Health Organization (WHO) analysis, ovulation occurs from 24 to 56 h after the onset of the LH surge, with a mean time of 32 h (World Health Organization 1980). However the surveillance of sperm in the cervix is up to 80 h after intercourse (Gould et al. 1984). A 2010 systematic review of trials that evaluated the effectiveness of different synchronization methods in stimulated and natural cycles for IUI in subfertile couples concluded the choice should be based on hospital facilities, medical staff, convenience for the patient, costs and drop-out levels as no method was clearly superior to another (Cantineau et al. 2010).

In this study, we aimed to evaluate the impact of IUI timing on pregnancy rates in clomiphene citrate/IUI cycles.

## Methods

The protocol for the research project has been approved by the Ethics Committee of Zekai Tahir Burak Women’s Health Education and Research Hospital within which the work was undertaken and it conforms to the provisions of the Declaration of Helsinki (as revised in Tokyo 2004). The medical records of 280 infertile patients who have demonstrated at least one patent fallopian tube on hysterosalpingography and whose partners had normal spermiogram analysis results based on World Health Organization (WHO) 2010 criteria have been reviewed after excluding patients with endometriosis, medical comorbidities and hormonal disturbances. All patients have received clomiphene citrate treatment (50–150 mg/day orally starting on 3–5 cycle day of menstruation and lasting on 7–9 cycle days of menstruation) for ovulation induction followed by one intrauterine insemination procedure performed on 24 or 36 h after ovulation trigger by exogenous 10,000 IU intramuscular urinary hCG injection upon detection of a mature follicle with ≥17 mm diameter. Selection of daily dose of CC was decided by regarding the lowest effective dose which induces at least one dominant follicle during ovulation induction. The IUI attempts of the whole study group was ≤6 and ≤3 for PCOS and unexplained infertile patients respectively due to the ovulation induction policy of our institution by using oral CC treatment. The demographic features, infertility types, dominant follicle number, endometrial thickness on hCG day, hCG type (recombinant or urinary derived hCG) timing of intrauterine insemination and clinical pregnancy rates of the patients have been evaluated. Following the IUI procedures, the patients have not received vaginal progesterone supplementation for luteal support. A clinical pregnancy was defined as the presence of a gestational sac with accompanying fetal heartbeat by ultrasound at least 4 weeks after IUI. Statistical analysis was performed by using IBM SPSS Statistics Software (17.0, SPSS Inc., Chicago, IL, USA). Kolmogorov–Smirnov test has been used for evaluating the normality of the distributions of continuous variables. The parametric results were presented as mean ± standard deviation values and normal distributed values were compared by using the independent samples t test. Comparison of nonparametric data and parametric data without normal distribution have been made by using Mann–Whitney U test. Categorical variables were compared with Fisher’s exact or Pearson Chi square tests. p values <0.05 were considered statistically significant.

## Results

**Fig. 1:**
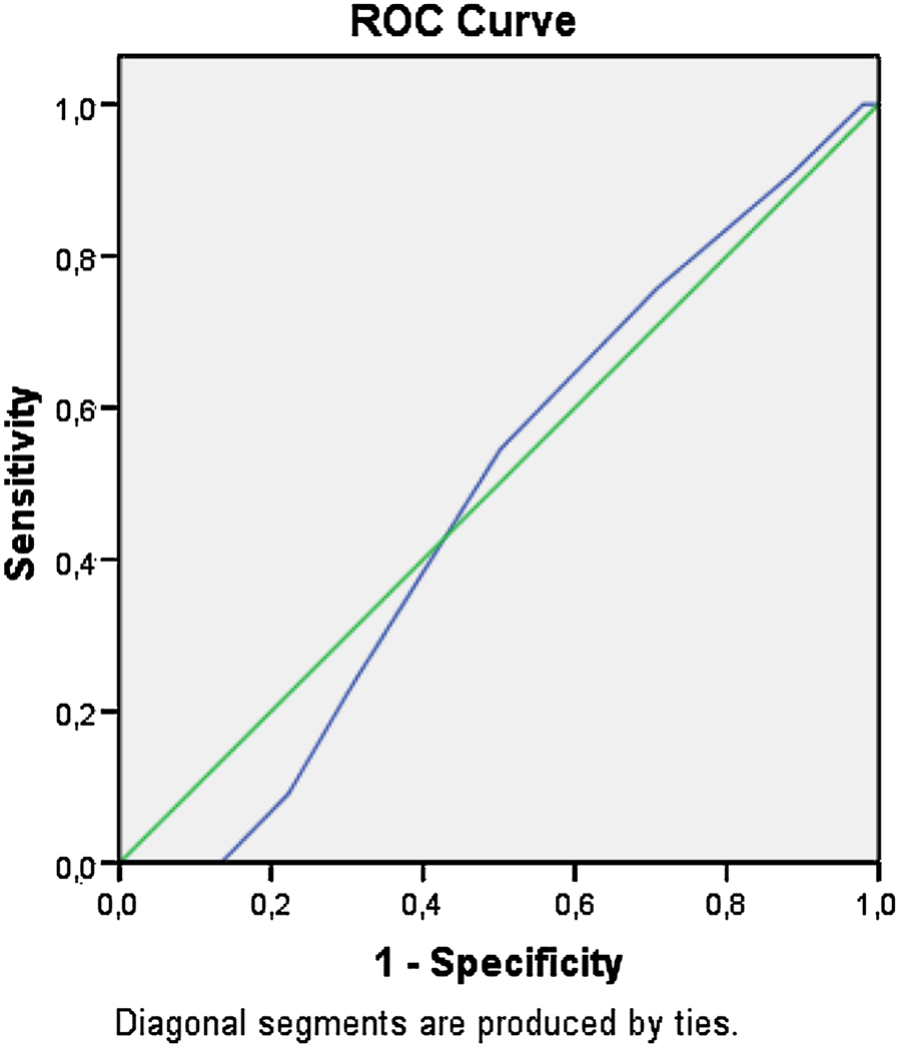
Receiver operating characteristic (ROC) analysis of the mature follicle diameter and clinical pregnancy rate

**Fig. 2:**
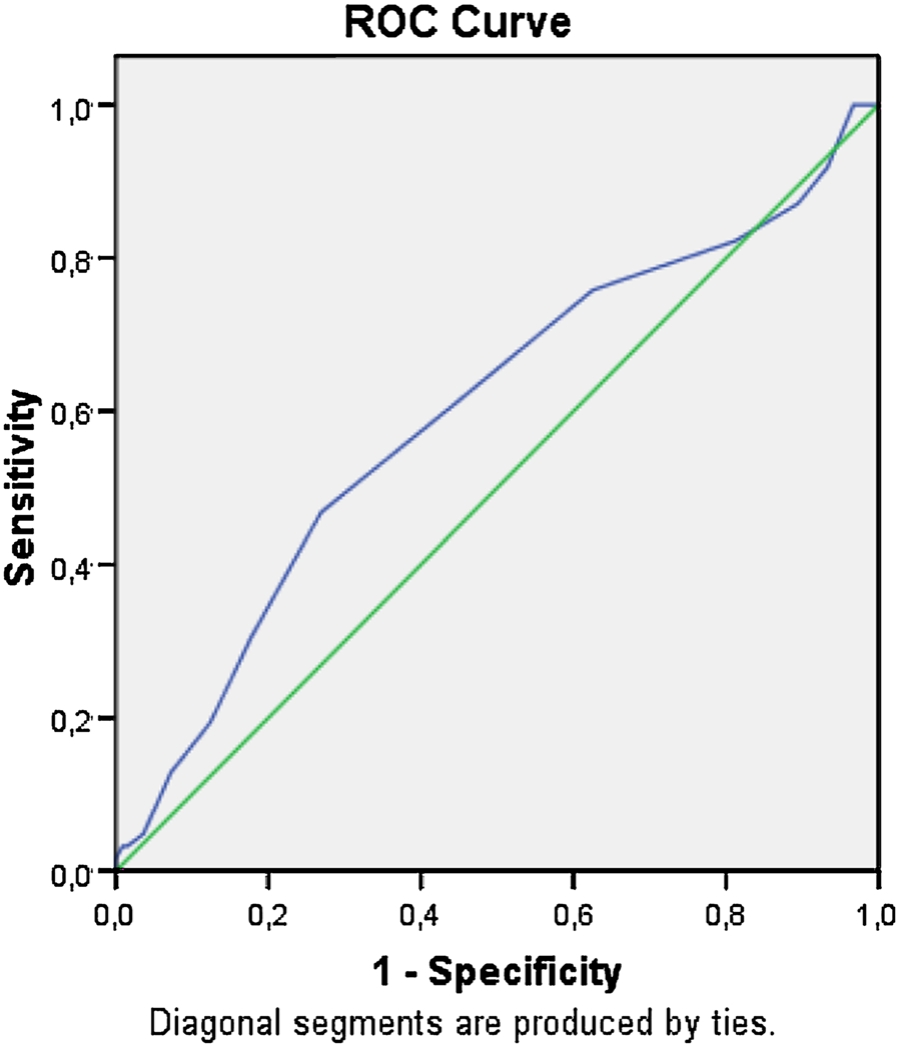
Receiver operating characteristic (ROC) analysis of delayed hCG trigger during ovulation induction treatment and clinical pregnancy rate. Area under curve (AUC) 0.59; 95 % CI 0.51–0.67; p 0.01

The age, basal (cycle day 3) FSH, mean mature follicle number and infertility duration was found as 26.4 ± 4.9, 6.6 ± 1.6, 1.55 ± 0.6 and 3.3 ± 2.0, respectively. Thirty-nine percent of the patients (N:110) have been diagnosed as polycystic ovary syndrome (PCOS) and 61 % of the patients (N:170) have been diagnosed as unexplained infertile. Clinical pregnancy rate per cycle was 12.7 % for PCOS patients and 11.2 % for unexplained infertile patients (p:0.69). The clinical pregnancy rate of the study group based on the timing of intrauterine insemination (24 vs. 36 h following hCG trigger) was found to be similar regardless of infertility type (p:0.97). Receiver Operating Characteristic (ROC) analysis of the mature follicle diameter and clinical pregnancy rate was found to be statistically nonsignificant (ROC AUC:0.48; p:0.79) (Fig. 1). The cycle day of which hCG trigger has been performed was found to be longer for patients who have achieved clinical pregnancy than patients who have not got pregnant following the treatment cycle (mean cycle day number: 14.1 ± 2.9 vs. 13.3 ± 2.5; ROC AUC:0.59; p:0.01) (Fig. 2).

## Discussion

Intrauterine insemination with controlled ovarian hyperstimulation has been used over the years, as a treatment for mild male factor, anovulation and unexplained infertility (Goverde et al. 2000; Guzick et al. 1999). The time of administration of intrauterine insemination is the most critical factor. Regimens vary between centers and also between clinicians. Hence the correct timing of insemination to improve pregnancy is recently a debated issue.

Pryor et al. showed an improved pregnancy rate when IUI was performed with a time interval of 38–40 h (60 % pregnancy rate) as compared to shorter IUI interval (0 % pregnancy rate) after hCG. However, the small number of patients and the different sperm preparation techniques used between the two groups were the main limitations of the study. While 54 % of patients in the 38–40 h group had sperm prepared with a sperm swim-up technique, only 13 % of those in the 32–34 h group had this type of sperm preparation. Also the males in the Pryor study were all with spinal cord injury and had sperm recovered via vibratory stimulation. The sperm counts were low and the sperm preparation techniques may have been more of a deciding factor than was the timing of the IUI and may have accounted for the difference in pregnancy rates (Pryor et al. 2001).

There are some important factors affecting the outcome of COH-IUI. These factors include female age, tube malfunction, duration of infertility, endometrial thickness, number of mature follicles, various techniques of semen preparation, sperm motility and concentration (Campana et al. 1996; Duran et al. 2002; Khalil et al. 2001; Demir et al. 2011; Tomilson et al. 1996). In our study, we found no significant difference with respect to these parameters affecting the outcome among the groups. Also there was no relationship between the average mature follicle size and clinical pregnancy outcomes (ROC AUC:0.48; p:0.79). In this study, we have demonstrated that the longer the treatment cycle with clomiphene citrate, the higher the clinical pregnancy rate. This result could have been related to the delayed maturity of dominant follicles during clomiphene citrate treatment cycles. Patiently waiting for later hCG trigger during ovulation induction with CC followed by intrauterine insemination or detection of spontaneous LH surge on urine LH ovulation kit can potentially increase the clinical pregnancy rates regardless of the timing of intrauterine insemination.

Robb et al. (2004) showed no significant difference in pregnancy outcomes between IUIs performed at 24 versus 36 h after hCG with 182 clomiphene citrate/IUI cycles in 90 women. However the small numbers, low overall pregnancy rates and inclusion of various causes of female infertility diagnosis, including poor ovarian reserve, anovulatory, unexplained, anatomic, male factor are the possible weaknesses of the retrospective study. In our study the patients were divided in two groups. One hundred and ten (39 %) patients were polycystic ovary and one hundred and seventy (61 %) patients were unexplained. To homogenize the study groups, we excluded other possible causes of infertility. Clinical pregnancy rates per cycle was 12.7 % in PCOS group and 11.2 % was in unexplained group (p:0.69). There was also no significant difference in pregnancy outcomes whether hCG was administered at 24 or 36 h prior to IUI in clomiphene citrate stimulated cycles in both groups (p:0.97). Our results were consistent with Lewis, Wang and Claman’s (Lewis et al. 2006; Claman et al. 2004). In an other study, Ragni et al. (2004) showed significant increases in pregnancy rates when intrauterine insemination was performed during the preovulatory and periovulatory periods, but not the postovulatory period. In a retrospective study, Xu et al. have concluded that the female age, infertile duration, ovarian stimulation and follicle number, cause of infertility were the main factors affecting clinical pregnancy outcome. In the same study, the sperm density, and cycle numbers have been found to influence pregnancy outcome too but the insemination timing, and frequency have been found to exert little effect (Xu et al. 2013). In a prospective randomized trial, Rahman et al. (2010) have evaluated the clinical efficacy of double IUI over single IUI and no benefit of double IUI has been detected among unexplained infertile patients. Although the best application time and frequency for IUI procedures have not been detected, longer survival period of spermatozoa within the female genital tract, unlike oocyte, result with broader time interval preferences for successful IUI procedures. Future studies are needed for clarifying the best timing for IUI procedures to enhance pregnancy rates following ovulation induction.

To conclude, our data showed intrauterine insemination can be done successfully at either 24 or 36 h after hCG in clomiphene citrate stimulated cycles. This will allow more flexibility and convenience for both physicians and patients, especially for unnecessary obligation to perform IUI procedures on weekends.

## Conclusion

In this study, our data showed intrauterine insemination timing did not affect the cycle outcomes whether the procedure has been performed 24 or 36 h later following ovulation trigger with exogenous hCG utilization. The longer period of treatment cycle during ovulation induction with clomiphene citrate resulted with higher clinical pregnancy rate. Intrauterine insemination can be done successfully at either 24 or 36 h after hCG in clomiphene citrate stimulated cycles. This will allow more flexibility and convenience for both physicians and patients, especially during weekends.
